# Supplementary material for: Patterns of Intron Gain and Loss in Fungi
Source: PLoS Biol. 2004 Nov 30;2(12):e422. doi: 10.1371/journal.pbio.0020422 (PMC532390; doi:10.1371/journal.pbio.0020422)
Supplement: Table S1 — Also available at http://genes.mit.edu/NielsenEtAl/. (4.3 MB ZIP). [file pbio.0020422.st001.zip › NielsenEtAl/html/796.html]

AN1953.1.NCU04247.1.MG09547.1.FG10385.1


```
 CLUSTAL W (1.82) Multiple Sequence Alignments - Introns Inserted


Sequence 1: NCU04247.1	380 aa
Sequence 2: FG10385.1	380 aa
Sequence 3: MG09547.1	393 aa
Sequence 4: AN1953.1	380 aa
Alignment Length: 394 aa
Number Identitical Residues: 278 aa
Alignment Score (without introns) 12196


MG09547.1 	MAQL-LHNSPIVIDNGSGTIRAGFAGEDLPKCFFPSFVGRPKHLKVLAGALEGDVFIGEK
NCU04247.1	MTDS-LHNAPIVLDNGSGTIRAGFAGDDVPKCHFPSFVGRPKHLRVLAGALEGEVFIGQK
FG10385.1 	MADS-LHNAPIVLDNGSGTIRAGFAGDDLPKCFFPSWVGRPKHLRVLAGALEGEVFIGQK
AN1953.1  	MTEATLHNAPIVIDNGSGTIRAGFAGEEIPSCYFPSFVGRPKHPRVMAGGLEGDVFIGQR
          	*:: :***:***:*************:::*.*.***:****** :*:**.***:****::

MG09547.1 	AATELRGLLKIRYPLEHGIVTDWDDMEKIWGYVYEEGLKVLSEE~VSLHRSWPPRPR0HP
NCU04247.1	AASELRGLLKIRYPLEHGIVTDWDDMEKIWAYVYDEGLKTLSEE0------------~HP
FG10385.1 	AASELRGLLKIRYPLEHGIVTDWDDMERIWEYVYGEGLKTLSEE0------------~HP
AN1953.1  	-AQELRGLMKIRYPLEHGIVTDWDDMEKIWHYVYENELKTLPEE~------------~HP
          	 * *****:******************:** *** : **.*.**              **

MG09547.1 	VLLTEPPLNPRANRDTAAQILFETFNVPALYTSIQAVLSLYASGRTTGIVLDSGDGVSHA
NCU04247.1	VLLTEPPLNPRANRDTAAQILFETFNVPALYTSIQAVLSLYASGRTTGVVLDSGDGVSHA
FG10385.1 	VLLTEPPLNPRSNRDTAAQILFETFNVPALHTSIQAVLSLYASGRTTGIVLDSGDGVSHA
AN1953.1  	VLLTEPPLNPRKNRDIAAQIMFETFNVPALYTSIQAVLSLYASGRTTGVVLDSGDGVSHA
          	*********** *** ****:*********:*****************:***********

MG09547.1 	VPVYEGFAITNSIQRIDVAGRDVTEHLQTLLRKSGYVFHTSAEKEVVRLIKEATSYVAHD
NCU04247.1	VPVYQGFTVPNSIRRIDVAGRDVTEYLQTLLRKSGYVFHTSAEKEVVRLIKESVTYVAHD
FG10385.1 	VPVYEGFAMPSSIRRIDVAGRDVTEYLQTLLRKSGYVFHTSAEKEVVRLIKESVSYVAHD
AN1953.1  	VPVFEGFAIPNSIRRIDVAGRDVTEQLQLLLRKTGHVLHTSAEKEVVRMIKEKVCYVSLD
          	***::**::..**:*********** ** ****:*:*:**********:*** . **: *

MG09547.1 	PKKEEKEWAGAKLDPSKMMSDYTLPDGNKIK0LGAERFRAPEILFDPEIIGLEYPGVHQM
NCU04247.1	PRKEEKEWAAAKMDPAK-IAEYVLPDGNKLK0IGAERFRAPEILFDPEIIGLEYPGVHQI
FG10385.1 	PRKEERDWVGVKPNDSK-VAEYVLPDGYKLK0IGAERFRAPEILFDPEIIGLEYPGVHQI
AN1953.1  	PKREEKEWMNSYKSESK-HADYVLPDGHKIK0IGQERFRAPEILFDPELIGLEYPGVHQI
          	*::**::*     . :*  ::*.**** *:* :* *************:**********:

MG09547.1 	VINAIGKTDLDLRKSLYSNIVLSGGSTLTKGFGDRLLTEVQKLAVRDMRIKIFAPPERKY
NCU04247.1	VVDSINRTDLDLRRDLYSNIVLSGGSTLTKGFGDRLLTEVQKLAVKDMRIKIFAPPERKY
FG10385.1 	VVDAINRTDLDLRKSLYSNIVLSGGSTLTKGFGDRLLTELQKLAVKDMRIKIFAPPERKY
AN1953.1  	VQDAIIRTDLDLRKSLYLNIVLSGGSTLCKNFPDRLMREIKRLAVEDMKIRISAPAERKY
          	* ::* :******:.** ********** *.* ***: *:::***.**:*:* **.****

MG09547.1 	STWIGGSILAGLSTFRK0MWVSMDDWHENPDIIHTKFN
NCU04247.1	STWIGGSILAGLSTFRK0MWVSIDDWHENPDIIHTKFT
FG10385.1 	STWIGGSILAGLSTFRK0MWVSIDDWHENPDIIHTKFT
AN1953.1  	TTWIGGGILAGLSTFRK0MWVSADEWHEDPEIIHRKFA
          	:*****.********** **** *:***:*:*** **
```
